# Overlooked Carbamazepine Toxicity: A Diagnostic Pitfall in an Elderly Patient With Dizziness

**DOI:** 10.7759/cureus.78802

**Published:** 2025-02-10

**Authors:** Koki Kobayashi, Ayano Hamai, Takayuki Ando

**Affiliations:** 1 Department of General Medicine, Awa Regional Medical Center, Chiba, JPN; 2 Center for General Medicine Education, School of Medicine, Keio University, Tokyo, JPN

**Keywords:** adverse drug reactions, auto induction, carbamazepine (cbz), carbamazepine toxicity, diagnostic error, dizziness, drug induced dizziness

## Abstract

Dizziness is a common chief complaint that often poses a diagnostic challenge. A 79-year-old man was initially misdiagnosed with peripheral vertigo based on normal neuroimaging but later developed altered consciousness and involuntary limb movements, ultimately revealing carbamazepine (CBZ) toxicity. This case highlights a critical diagnostic pitfall: prematurely attributing dizziness to peripheral vertigo without considering drug-induced syndromes. Although the potential role of concomitant amiodarone use remains uncertain, this case also underscores the importance of cautious CBZ dose escalation, serum level monitoring, and a thorough medication review. Recognizing CBZ toxicity as a mimicker of central vertigo is crucial for ensuring timely and accurate diagnosis, especially in elderly patients.

## Introduction

Dizziness is a common complaint among the elderly [1]. Previous studies suggest that 23% of dizziness cases in patients aged 65 and older are attributed to adverse drug reactions. Among those elderly patients presenting with dizziness, approximately 33% use more than five medications, and 37% take three or more drugs that increase fall risk [2]. Drugs induce dizziness by various mechanisms [3], including inner ear damage, impaired cerebral circulation, and central nervous system (CNS) depression. Medications such as aminoglycosides, loop diuretics, and salicylates can cause inner ear damage, while antihypertensives may impair cerebral circulation. CNS depressants, including antiepileptics, anxiolytics, and antipsychotics, can also cause dizziness. Distinguishing drug-induced dizziness from vestibular and cardiovascular causes is challenging, especially in elderly patients with polypharmacy, as symptoms often overlap.

Carbamazepine (CBZ) is an antiepileptic drug widely used to treat neuropathic pain, including trigeminal neuralgia. Rapid dose escalation or drug interactions can result in elevated serum levels and toxicity [4]. Herein, we report a case of CBZ toxicity in an elderly patient, due to a rapid dose increase, and discuss the importance of differentiating drug-induced vertigo from other causes.

## Case presentation

A 79-year-old man presented to the Emergency Department (ED) with altered consciousness and involuntary limb movements. Eleven days prior to admission, he had been prescribed CBZ 200 mg/day for trigeminal neuralgia. Nine days before admission, he reported persistent dizziness and visited the ED. Although the general examination and gait assessment were unremarkable, the finger-to-nose test revealed poor performance, raising suspicion of central vertigo. Consequently, head imaging was performed to further evaluate the cause. Computed tomography (CT) scans and magnetic resonance imaging (MRI) of the brain did not show any signs of acute stroke or metabolic encephalopathy. He was diagnosed with peripheral vertigo and discharged.

**Figure 1:**
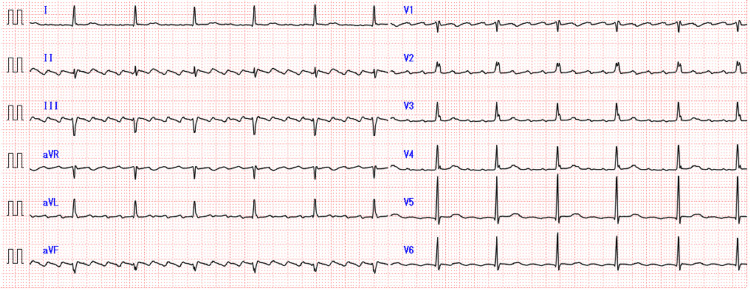
ECG of 4:1 conduction atrial flutter recorded at the ED aVL: augmented vector left; aVR: augmented vector right; aVF: augmented vector foot; ECG: electrocardiogram; ED: emergency department

Four days prior to admission, his CBZ dose was increased to 800 mg/day because of severe facial pain. Three days prior to admission, he returned to the ED with presyncopal dizziness, exacerbated by standing and exertion. An electrocardiogram (ECG) showed atrial flutter, with a heart rate of 71 beats per minute and 4:1 conduction (Figure 1). This easily changed to 2:1 conduction with exertion, exacerbating presyncopal dizziness. Electrical cardioversion was performed, resolving the arrhythmia. He was admitted to cardiology, started on amiodarone 400 mg/day, and discharged three days later.

**Figure 2:**
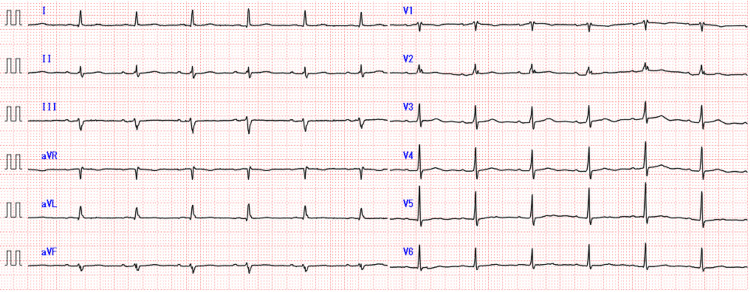
ECG on admission showing normal sinus rhythm with no significant abnormalities detected aVL: augmented vector left; aVR: augmented vector right; aVF: augmented vector foot; ECG: electrocardiogram

On the discharge night, his wife noticed that the patient’s mental status had altered and involuntary limb movements; they visited the ED again. Upon examination, the vital signs were stable: temperature of 37.0℃, blood pressure of 152/78 mmHg, heart rate of 74 bpm, respiratory rate 20/min, and oxygen saturation 97% on room air. The patient demonstrated mild disorientation, mistaking the date for one day. Pupillary light reflexes were 3+ bilaterally, with no facial paralysis, sensory disturbances, or muscle weakness. Babinski’s sign was negative, and cerebellar ataxia was absent. Myoclonic-like contractions were observed in all limbs. No jugular venous distention, neck stiffness, murmurs, tachycardia, wheezing, or rales were noted. The abdomen was slightly distended, and no lower extremity edema was present. Current medications included CBZ 200 mg tablets, two tablets twice daily (total 800 mg/day), amiodarone 400 mg/day, edoxaban 30 mg/day, tramadol-acetaminophen, bisoprolol 1.25 mg/day, and pregabalin 50 mg/day. ECG was unremarkable (Figure 2).

Referring to the laboratory results (Table 1), the serum CBZ concentration was markedly elevated at 18.8 μg/mL (reference range: 4-12 μg/mL), strongly indicative of CBZ toxicity. The mild elevation of B-type natriuretic peptide (BNP) (22.6 pg/mL, reference range: <18.4 pg/mL) does not support a diagnosis of concurrent heart failure. The metabolic panel revealed no elevation in liver transaminases, and there were no abnormalities in electrolytes or thyroid function.

**Table 1:** Blood investigations of the patient on admission to the ED The elevated CBZ concentration (18.8 μg/mL, reference range: 4-12 μg/mL) confirms CBZ toxicity. Other results, including liver function and electrolytes, were within normal limits, ruling out alternative metabolic or hepatic causes. GOT (AST): glutamate oxaloacetate transaminase (aspartate aminotransferase); GPT (ALT): glutamate pyruvate transaminase (alanine aminotransferase); LDH: lactate dehydrogenase; ALP: alkaline phosphatase; γ-GTP: gamma-glutamyl transferase; CPK: creatine phosphokinase; BNP: B-type natriuretic peptide; eGFR: estimated glomerular filtration rate; CBZ: carbamazepine; ED: emergency department

Test	Result	Reference Range
White Blood Cell Count	7,300/μL	3,100-8,400/μL
Hemoglobin	14.2 g/dL	13.4-17.1 g/dL
Platelet Count	218 x 10^9/L	153-346 x 10^9/L
Total Protein	6.7 g/dL	6.6-8.1 g/dL
Albumin	3.8 g/dL	4.1-5.1 g/dL
Total Bilirubin	0.7 mg/dL	0.2-1.2 mg/dL
GOT (AST)	22 U/L	5-37 U/L
GPT (ALT)	17 U/L	6-43 U/L
LDH	214 U/L	124-222 U/L
ALP	62 U/L	38-113 U/L
γ-GTP	29 U/L	<75 U/L
CPK	147 U/L	57-240 U/L
Blood Urea Nitrogen	20 mg/dL	9-21 mg/dL
Creatinine	0.81 mg/dL	0.65-1.09 mg/dL
Sodium	140 mEq/L	138-145 mEq/L
Chloride	103 mEq/L	98-107 mEq/L
Potassium	4.7 mEq/L	3.5-5.0 mEq/L
Thyroid-Stimulating Hormone	0.66 μIU/mL	0.6-4.2 μIU/mL
Lactate	2.4 mmol/L	0.5-2.2 mmol/L
BNP	22.6 pg/mL	<18.4 pg/mL
eGFR	69.4 mL/min/1.73 m^2^	>60 mL/min/1.73 m^2^
Serum CBZ Concentration	18.8 μg/mL	4-12 μg/mL

He was admitted to the hospital, stopped CBZ, and received intravenous fluids. His mental status and involuntary movements improved significantly by the second day, and neurological symptoms had completely resolved by day 4. As his trigeminal neuralgia symptoms also improved without the need for analgesics during hospitalization, CBZ was permanently discontinued, and no alternative pain medications were prescribed. He was discharged home with a plan for outpatient follow-up to monitor for the potential recurrence of symptoms.

## Discussion

This case highlights the importance of differentiating between peripheral vertigo, central vertigo, and drug-induced toxicity, particularly CBZ toxicity. In this case, the delayed diagnosis of CBZ toxicity can be attributed to a cognitive error known as premature closure. This type of diagnostic error occurs when a diagnosis is accepted without thorough verification [5]. Initially, the absence of CNS lesions on imaging led to the premature conclusion that the patient's symptoms were due to peripheral vertigo. Compounding this error, during the patient's subsequent visit, the presence of atrial flutter further misled clinicians toward a diagnosis of cardiogenic presyncope, overshadowing the possibility of drug-induced dizziness, particularly CBZ toxicity.

Peripheral vertigo typically presents with episodic dizziness triggered by head movements, often accompanied by auditory symptoms like tinnitus or hearing loss, without significant neurological deficits. Central vertigo, on the other hand, involves continuous dizziness and is frequently associated with neurological signs such as ataxia, dysarthria, and abnormal eye movements. CBZ toxicity can mimic central vertigo, causing persistent dizziness, ataxia, involuntary movements, nystagmus, and altered consciousness. These overlapping symptoms can complicate the differentiation between the two. The ATTEST method - focusing on Associated Symptoms, Timing, Triggers, Examination Signs, and Testing - emphasizes identifying associated symptoms, timing, and triggers [6]. Triggers are helpful in differentiating drug-induced vertigo. We summarize the key features of these three conditions in Table 2.

**Table 2:** Differentiation of peripheral vertigo, central vertigo, and CBZ toxicity This table compares key features of peripheral vertigo, central vertigo, and CBZ toxicity. It highlights differences in symptoms, triggers, and physical findings, helping clinicians distinguish CBZ toxicity from other causes of dizziness. CT: computed tomography; MRI: magnetic resonance imaging; CBZ: carbamazepine

Types of Vertigo	Peripheral Vertigo	Central Vertigo	Carbamazepine Toxicity (Drug-Induced Vertigo)
Associated Symptoms	Tinnitus, hearing loss, ear fullness, nausea	Neurological symptoms (paralysis, numbness, speech impairment, diplopia), nausea, headache	Fatigue, drowsiness, nausea, confusion, ataxia, decreased consciousness, arrhythmia, syncope
Onset	Acute, episodic	Sudden or gradual, persistent	Acute, persistent
Triggers	Positional changes (head or body)	No specific triggers	Dose increase, addition of other medications
Physical Findings	Horizontal nystagmus, direction-fixed nystagmus, mild balance disturbance, inner ear impairment	Vertical or direction-changing nystagmus, skew deviation, diplopia, dysarthria, dysphagia, dysphonia, dysmetria, dysdiadochokinesis	Vertical nystagmus, myoclonus, delayed reactions, impaired consciousness, seizures, diplopia, dysarthria, dysphagia, various other neurological findings
Additional Tests	Hearing tests, caloric testing, positional nystagmus tests (vestibular function tests)	Brain MRI, CT, neurological examinations	Blood carbamazepine level testing; blood tests for liver/kidney function, and electrolyte

CBZ inhibits neuronal depolarization by blocking sodium channels in the CNS and is commonly used for neuropathic pain. Its anticholinergic effects can cause nausea, constipation, and urinary retention, while sodium channel blockade may lead to cardiovascular issues such as QRS prolongation, arrhythmias, and hyponatremia [4]. CBZ causes non-epileptic myoclonus, tics, and ataxia [7]. Dizziness caused by CBZ is often accompanied by ataxia, involuntary movements, and vertical nystagmus, which are key signs of central vertigo and require careful differentiation.

In elderly patients, reduced CYP3A4 activity increases the risk of CBZ toxicity. Gabapentin, with fewer CNS side effects and minimal drug interactions due to the lack of CYP3A4 induction, is a safer alternative, especially for those on polypharmacy [8].

CBZ is metabolized by CYP3A4 and induces its own metabolism (auto-induction). During the initial phase of treatment or after dose adjustments, the drug’s half-life may be prolonged, leading to rapid increases in blood concentration. In this case, the patient’s symptoms appeared to be associated with a rapid increase in CBZ dosage after initiation.

Although no direct studies have established a clear relationship between CBZ and atrial flutter, sodium channel blockers are known to suppress atrial conduction [9]. In this case, CBZ may have contributed to atrial flutter by prolonging the flutter cycle length. Further studies are needed to clarify the potential role of CBZ in atrial flutter development.

CBZ is also well-known for its numerous drug interactions; therefore, careful attention is required when administering drugs metabolized by the same pathway, such as antiviral and antifungal agents [10]. Amiodarone, which was administered in this case, is also metabolized by CYP3A4. Although there are reports that amiodarone does not affect the blood concentration of CBZ [11], it is better to monitor blood levels carefully. It is crucial to check for signs of toxicity when physicians find CBZ on a patient's medication list.

## Conclusions

Central vertigo and CBZ toxicity share similar features, such as persistent dizziness and acute-onset neurological symptoms. Therefore, CBZ toxicity should be considered in elderly patients with dizziness and neurological symptoms, along with a thorough medication review.

This case underscores the risks of CBZ dose escalation in elderly patients, particularly due to reduced CYP3A4 activity and CBZ auto-induction, which can lead to rapid toxicity. In this population, careful dose titration and drug interaction monitoring are essential to prevent adverse effects.
